# Subretinal delivery of erythropoietin alleviates the N-methyl-N-nitrosourea-induced photoreceptor degeneration and visual functional impairments: an *in vivo* and *ex vivo* study

**DOI:** 10.1080/10717544.2017.1370620

**Published:** 2017-09-09

**Authors:** Ye Tao, Yue Wang, Zhao Ma, Liqiang Wang, Limin Qin, Lu Wang, Yi Fei Huang, Shizhong Zhang

**Affiliations:** a Department of Ophthalmology, Key Lab of Ophthalmology and Visual Science, Chinese PLA General Hospital, Beijing, PR China;; b The National Key Clinical Specialty, The Engineering Technology Research Center of Education Ministry of China, Guangdong Provincial Key Laboratory on Brain Function Repair and Regeneration, Department of Neurosurgery, Zhujiang Hospital, Southern Medical University, Guangzhou, PR China;; c Department of Neurosurgery, Central Hospital of Wuhan, Tongji Medical College, Huazhong University of Science and Technology, Wuhan, PR China

**Keywords:** Neurodegeneration, erythropoietin, subretinal, drug delivery, protection

## Abstract

Retinitis pigmentosa (RP) is a heterogeneous group hereditary retinal disease that is characterized by photoreceptor degeneration. The present study sought to explore the therapeutic effects of erythropoietin (EPO) on the N-methyl-N-nitrosourea (MNU)-induced photoreceptor degeneration. The MNU-administered mouse or normal control received a subretinal injection of EPO (at the dose of 10U). Twenty-four hours after EPO injection, the retinal EPO levels of experimental animals were quantified. Subsequently, the experimental animals were subjected to optokinetic tests, ERG examination, SD-OCT examination, histology assessment, and immunohistochemistry evaluation. The retinal superoxide dismutase (SOD) activity, malondialdehyde (MDA) content, and expression levels of several apoptotic factors were also quantified. The subretinal injection of EPO up-regulated the retinal EPO level in the retinas of MNU-administered mice. The optokinetic tests and ERG examination suggested the visual functional impairments in MNU-administered mice were ameliorated after EPO treatment. The SD-OCT and histological examination suggested the morphological devastations in MNU-administered mice were alleviated after EPO treatment. The cone photoreceptors in MNU-administered mice were protected from the MNU-induced detrimental effects. Moreover, the EPO treatment rectified the apoptotic abnormalities in MNU-administered mice, and enhanced the expression level of Foxo3, a critical mediator of autophagy. The EPO treatment also mitigated the MDA concentration and enhanced the retinal SOD activity, thereby counteracting the retinal oxidative stress in MNU administered mice. In ophthalmological practice, the subretinal delivery of EPO is a feasible therapeutic strategy to alleviate photoreceptor degeneration. These findings would enrich our pharmacological knowledge about EPO and shed light on the development of an effective therapy against RP.

## Introduction

Photoreceptors in the outer nuclear layer (ONL) of retina are responsible for the perception of light and color. Retinitis pigmentosa (RP) is a heterogeneous group hereditary retinal disease that is characterized by the initially impaired dark-adapted sight, progressive deterioration of visual fields, and eventual blindness (Geneva II, 2016; Moskowitz et al., 2016). Although remarkable advancements have been made in the therapeutic strategy against RP, the effects of existed clinical treatments remain far from satisfactory. The futility should be ascribed to the enormous heterogeneity implying in the etiology, since more than 180 gene mutations can initiate photoreceptor degeneration in RP (Tao et al., 2016). Currently, the specific molecular mechanism underlying RP is not thoroughly characterized. Sever lines of evidences suggest that the photoreceptor apoptosis is recognized as the primary pathological process of various RP phenotypes. It is noteworthy that the oxidative stress is related to the photoreceptor apoptosis in both RP patients and animal models (Yu & Cringle, 2005; Nowak, 2013). Oxidative insults can impair mitochondrion, perturb redox status, alter mitochondrial membrane permeability, and induce cytochrome c leakage. The leakage of cytochrome c from mitochondrion is considered as a pivotal initiator in apoptotic cascades. Moreover, Oxidative stress elevates excessively the poly adp-ribose polymerase (PARP) activity and interacts with the transcription factors such as nuclear factor-kB (NF-kB) and activator protein-1 (AP-1). Bursts of reactive oxidative species (ROS) can mediate photoreceptor apoptosis via the up-regulation of Bax protein, the down-regulation of Bcl-2 protein, and the activation of caspase families (Fernández-Sánchez et al., 2015; Kruk et al., 2015). Therefore, oxidative stress should be considered as an upstream target for therapeutics against RP. To corroborate this notion, several kinds of antioxidants are given to the RP patients and animal models to alleviate the photoreceptor degeneration (Pasantes-Morales et al., 2002; Fernández-Sánchez et al., 2015). These investigations suggest that the molecular with anti-oxidative potency might be developed into mutation-independent therapeutics against RP.

RP animal models are essential to attempt a variety of therapeutic approaches. The N-methyl-N-nitrosourea (MNU) administered mouse is a powerful tool to test candidate therapies (Tsubura et al., 2010). As a DNA alkylating agent, the MNU paralyzes the excision repair machinery within nucleus, and thereby inducing photoreceptor apoptosis in mammalian retinas. After a single MNU administration, active signs of massive photoreceptor degeneration, such as the decreased ONL width, the degraded electroretinogram (ERG) response, and the hyper-expressed apoptotic labeling, would occur within one week (Tao et al., 2015; Rösch et al., 2017). These characteristics faithfully mimic the features of RP. Therefore, the MNU administered mouse has served as a pharmacologically induced RP model with rapidly progressive dynamics.

As a glycoprotein, erythropoietin (EPO) critically modulates the erythropoiesis in fetal liver and adult kidneys. EPO also acts as a pleiotropic growth factor beyond erythropoiesis (Maiese et al., 2004). In the retina, EPO is secreted by ganglion cells and müller cells to maintain cellular homeostasis (Caprara & Grimm, 2012). Unlike some neuroprotective compounds with enormous molecular size, EPO can traverse the intact blood-retina barrier (BRB) at therapeutic concentrations. Emerging evidences suggest the exogenous EPO can protect retinal ganglion cells from acute ischemia-reperfusion injury, experimental glaucoma, and axotomy-induced degeneration (Luo et al., 2015). Furthermore, the exogenous EPO counteracts oxidative impairments and maintains mitochondrial dysfunction in the retinal pigment epithelial (RPE) cell (Gawad et al., 2009). In clinical practice, the beneficial effects of exogenous EPO have been reported in three patients with diabetic retinopathy (Li et al., 2010). Therefore, it is highly possible that the EPO could be developed into a effective drug against retinal degenerative diseases. However, whether the EPO exerts beneficial effects on the RP models remains to be clarified yet. Accordingly, we delivered EPO into the subretinal cavity of MNU administered mice. We showed that the subretinal delivery of EPO could ameliorate the photoreceptor degeneration as well as the visual function impairments in the MNU administered mice. Future refinements of our findings would shed light on the development of an effective therapeutic against RP.

## Materials and methods

### Animals and pharmacological administration

**Figure 1.**
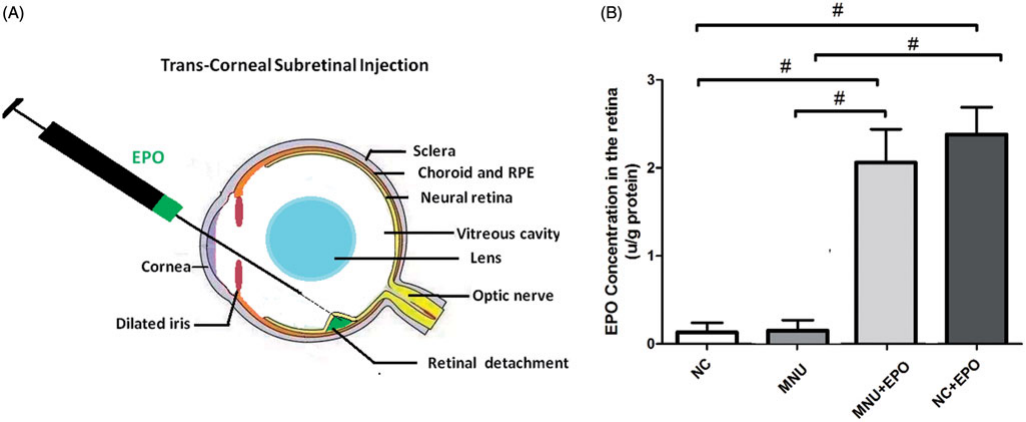
(A) The subretinal delivery pathway of EPO. A 30½-gague beveled needle was used to make an incision near the corneal limbus. The syringe needle of a Hamilton micro injector was inserted into the anterior chamber through the corneal perforation. The plunger of the Hamilton syringe was slowly pushed to deliver the EPO into the subretinal cavity. A successful subretinal injection would cause one or more retinal blebs on which retinal blood vessels were visible. (B) The retinal EPO level of the treated group was significantly higher than the normal controls. The retinal EPO level of the treated group was also significantly higher than the MNU group. The retinal EPO level of the Normal + EPO group was significantly higher than the normal controls (#*p* < .01 for differences compared between animal groups; All the values were presented as mean ± SD).

The C57BL/6 mice (male, 8 weeks old) were purchased from Laboratory Animal Center of General hospital of PLA (Beijing, China), and were housed in the experimental animal facility (room temperature: 18–23 °C, humidity: 40–65%) on a 12-h light/dark cycle. The care and maintenance of animals were performed in compliance with the ARVO guidelines for the Use of Animals in Ophthalmic and Vision Research. The animals were randomly assigned into four groups: (1) Normal control group: mice in this group were left without any pharmacological administration; (2) MNU group: mice in this group received the intraperitoneal administration of MNU (at the at the dose of 60 mg/kg; Sigma; St. Louis, MO); (3) EPO treated group: mice in this group received the subretinal injection of 10 U EPO (3 S Bio Co. Ltd., ShenYang, China) 1 h after MNU administration; (4) Normal control + EPO group: mice in this group received the subretinal injection of 10 U EPO. The subretinal injection followed the previous described methods (Petit et al., 2017). Briefly, the anesthetized mice were moved to an animal operating table under a microscope. A 30 ½-gague disposable beveled needle was used to make an incision near the corneal limbus (Figure 1(A)). The syringe needle of a Hamilton micro injector (Hamilton Company, Reno, NV) was inserted into the anterior chamber through the corneal perforation, avoiding the iris and lens to go deeper into the vitreous cavity and was stopped before the retinal inner surface. The plunger of the Hamilton syringe was slowly pushed to deliver the EPO into the subretinal cavity. After then, the syringe needle was carefully removed out and the neomycin/polymyxin B ophthalmic ointment (Xing Qi, Shenyang, China) was applied to the injected eyes to avoid further infection. Generally, a successful subretinal injection would cause one or more retinal blebs on which retinal blood vessels were visible. The complications of subretinal injection (such as cataracts and retinal puncture) were found in nine experimental animals. These mice were euthanized and were not involved in further evaluation.

### Enzyme-linked immunosorbent assay (ELISA) of retinal EPO level

Twenty-four hours after EPO treatment, the experimental animals were sacrificed and their eyecups were enucleated. The retina was isolated carefully and stored at –80 °C. Before detection, the retina tissue was homogenized in radio-immunoprecipitation assay (RIPA) lysis and extraction buffer, and then was sonicated at 0.5 Hz for 50 s (50-watt sonicator, Sonics & Materials, Danbury, CT). The retinal EPO concentration was evaluated by an ELISA kit (R&D Systems, Genetimes Technology, Inc., Shanghai, China) according to the manufacturer’s instructions. The protein levels of EPO in the retina were normalized by the total protein content, as determined by a bicinchoninic acid (BCA) kit. Absorbance at 450 nm and reference at 600 nm was measured by a microplate reader (Safire2, Tecan Group Ltd., Maennedorf, Switzerland).

### ERG recording

One week after MNU administration, the dark-adapted animals were anesthetized by an intraperitoneal injection of ketamine (80 mg/kg) and chlorpromazine (15 mg/kg, Jilin Shengda Animal Pharmaceutical Co., Ltd, China). Their pupils were dilated with 1% atropine and 2.5% phenylephrine hydrochloride (Xing Qi, Shenyang, China). Subsequently, they were transferred to the recording platform under dim red light. The top of the stage was fixed to the position where the animal eye faced the stimulus flash at a 20 cm distance. Their cornea was anesthetized with a drop of 0.5% proxymetacaine. The RETIport system (Roland Consult, Brandenburg, Germany) and custom-made chloride silver electrodes were used in the ERG recording. A loop electrode was placed over the cornea to serve as the active electrode. Needle reference and ground electrodes were inserted respectively into the cheek and tail. Scotopic ERGs were recorded at a stimulus intensity of 0.5 log cd-s/m^2^ with the inter-stimulus intervals of 30 s. Subsequently, the mice were light adapted for 10 min at the background intensity of 30 cd-s/m^2^. Photopic ERGs were recorded at the stimulus intensity of 1.48 log cd-s/m^2^ with the inter-stimulus intervals of 0.4 s. The band-pass (1 Hz-300 Hz) was used to amplify the recorded signals. The line noise was wiped off by a 50-Hz notch filter. Totally 60 photopic responses and 10 scotopic responses were collected and averaged for wave analysis. The amplitude of b-wave was defined as the distance between the trough and peak of each waveform.

### SD-OCT examination

After ERG recordings, the animals were transferred to the recording plane of the SD-OCT system (Bioptigen, Durham, NC) when they were still anesthetized. Their pupils were dilated with 1 atropine and 2.5% phenylephrine hydrochloride (Xing Qi, Shenyang, China). A corresponding box was centered on the Optic nerve head (ONH) with eight measurement points separated by 3 mm from each other. The SD-OCT cross-sectional images were analyzed with the InVivoVueTM DIVER version 2.4 software (Bioptigen, Inc., Morrisville, NC). The neural retinal thickness for examined eyes was compared at each point by measuring the distance from the vitreous face of the RGCs layer to the apical face of the RPE layer.

### Optokinetic testing

Light adapted visual acuity and contrast sensitivity of experimental animals were measured using a two-alternative forced choice paradigm as described previously (Umino et al., 2008). Briefly, stepwise functions for correct responses in both the clockwise and counter-clockwise direction were used to determine the response thresholds. The initial stimulus in visual acuity measurements was set as 0.200 cyc/deg sinusoidal pattern with a fixed 100% contrast. The initial pattern in contrast sensitivity measurements was set as 100% contrast, with a fixed spatial frequency of 0.128 cyc/deg. Contrast sensitivity was defined as 100 divided by the lowest percent contrast yielding a threshold response. All patterns were presented at a speed of 12 degrees/s with the mean luminance of 70 cd/m^2^. Visual acuities and contrast sensitivities of each mouse were measured for four times over a period of 24 h.

### Histology assessment and immunohistochemistry

The experimental animals were sacrificed and their eyecups were enucleated. Their eyecups were immersed in a fixative solution 4% paraformaldehyde (Dulbecco’s PBS; Mediatech, Inc., Herndon, VA) for 24 h. They were rinsed with PB (phosphate buffer), dehydrated in a graded ethanol series, and embedded in paraffin wax. Five sections with the thickness of 5 μm were cut vertically through the ONH of each eye. The sections were stained with hematoxylin and eosin (HE) and were evaluated by light microscopy. With the aid of the Image-Pro Plus software (Media Cybernetics, Silver Spring, MD), the adjacent thickness of the ONL was measured along the vertically superior–inferior axis at 250 μm intervals. The mean ONL thickness of each mouse was averaged from five sections. Then sections were rinsed in 0.01 M PBS, permeabilized in 0.3% Triton X-100, and blocked in 3% BSA for 1 h at room temperature. The sections were incubated overnight at 4 °C with a Alexa Fluor 488 conjugated peanut agglutinin (PNA, 1:200, L21409, Invitrogen, Carlsbad, CA), a rabbit polyclonal S-cone opsin antibody or a M-cone opsin antibody (1:400, Millipore, MA) that was diluted in 0.1% Triton X-100 and 1% BSA in PBS. The sections were extensively washed with PBS, and then incubated with Cy3-conjugated goat anti-rabbit IgG (1:400, 711-165-152, Jackson ImmunoResearch Laboratories, West Grove, PA) that was diluted in 0.01 M PBS . The sections were rapidly rinsed for five times with 0.01 M PBS, and then were covered with the anti-fade Vectashield mounting medium (Vector Laboratories, Burlingame, CA) for photographing. The Retinal whole mounts were prepared according to the previously described methods (Zhong et al., 2012). Briefly, the optic nerve bud and its surrounded sclera were cut from the back of the eyecup. Soft touching and gentle pressing by forceps on the sclera to help separate the whole neuroretinal layer from the RPE layer. Fluorescence in flat mounts was analyzed with the Zeiss LSM 510 META microscope (Zeiss, Thornwood, NY) fitted with Axiovision Rel. version 4.6 software (Carl Zeiss AGManufacturing company, Oberkochen, Germany). The number of cones presents within four 440 × 440 μm squares which located 1 mm superior to the center of the optic nerve was determined.

### Quantification of superoxide dismutase (SOD) activity and malondialdehyde (MDA) content

Three days post MNU administration, the animals were sacrificed and their eyecups were enucleated. The retinal tissue was added into the PBS containing 0.5% Triton X-100 (pH 7.4) and then was homogenized in ice cold by Grinders. The retinal tissue was centrifuged at 500 × *g* for 5 min at 4 °C. The suspension was assayed for protein content to normalize enzyme activity and content of MDA. The SOD activity was examined with the SOD Assay Kit-WST (Jiancheng Biotech Ltd., Nanjing, China). A spectrophotometer with ultra-micro cuvettes was used to measure the absorbance values. The content of MDA was assessed using a total bile acids colorimetric assay under the guidance of manufacturer’s instructions (Jiancheng Biotech Ltd., Nanjing, China).

### Quantitative reverse transcription-polymerase chain reaction (qRT-PCR)

Total RNA was extracted from pooled retinal patches with a commercial reagent (Trizol, Gibco Inc., Grand Island, NY), followed by cDNA synthesis using the μMACS DNA Synthesis kit (Miltenyi Biotech GmbH, Bergisch-Gladbach, Germany). Ten retinal patches from each animal group were prepared for qRT-PCR examination. Totally 40 retinal patches were involved in qRT-PCR examination. GAPDH was used as the internal standard of mRNA expression. Reactions were performed in a real-time CFX96 Touch PCR detection system (Bio-Rad Laboratories, Reinach, Switzerland). The amplification program consisted of polymerase activation at 95 °C for 5 min and 50 cycles of denaturation at 95 °C for 1 min, annealing and extension at 59 °C for 30 s. The primers used in qRT-PCR were: Bax: 5′-AGCTCTGAACAGATCATGAAGACA-3′ (forward) and 5′-CTCCATGTTGTTGTCCAGTTCATC-3′ (reverse); Bcl-2:5′-GGACAACATCGCTCTGTGGATGA-3′ (forward) and 5′-CAGAGACAGCCAGGAGAAATCAA-3′ (reverse); Caspase-3: 5′-TGTCGATGCAGCTAACC-3′ (forward) and 5′-GGCCTCCACTGGTATCTTCTG3′- (reverse); Calpian-2: 5′-CCCCAGTTCATTATTGGAGG3′ (forward) and 5′-GCCAGGATTTCCTCATTCAA-3′ (reverse); Foxo-3: 5′-CGGGATCCATGGCAGAGGCACCGGCTTC-3′ (forward) and 5′-GCTCTAGATCAGCCTGGCACCCAGCTCTG-3′ (reverse). The relative expression levels were normalized and quantified to obtain the ΔΔCT values (DATA assist Software version 2.2; Applied Biosystems, Foster City, CA).

### Statistical analysis

The statistical difference between different animal groups was processed using the ANOVA analysis followed by Bonferroni’s post-hoc analysis. *p* < .05 was considered significant. The values are presented as mean ± standard deviation (SD).

## Results

### Subretinal injection of EPO ameliorated the MNU-induced visual impairments

**Figure 2.**
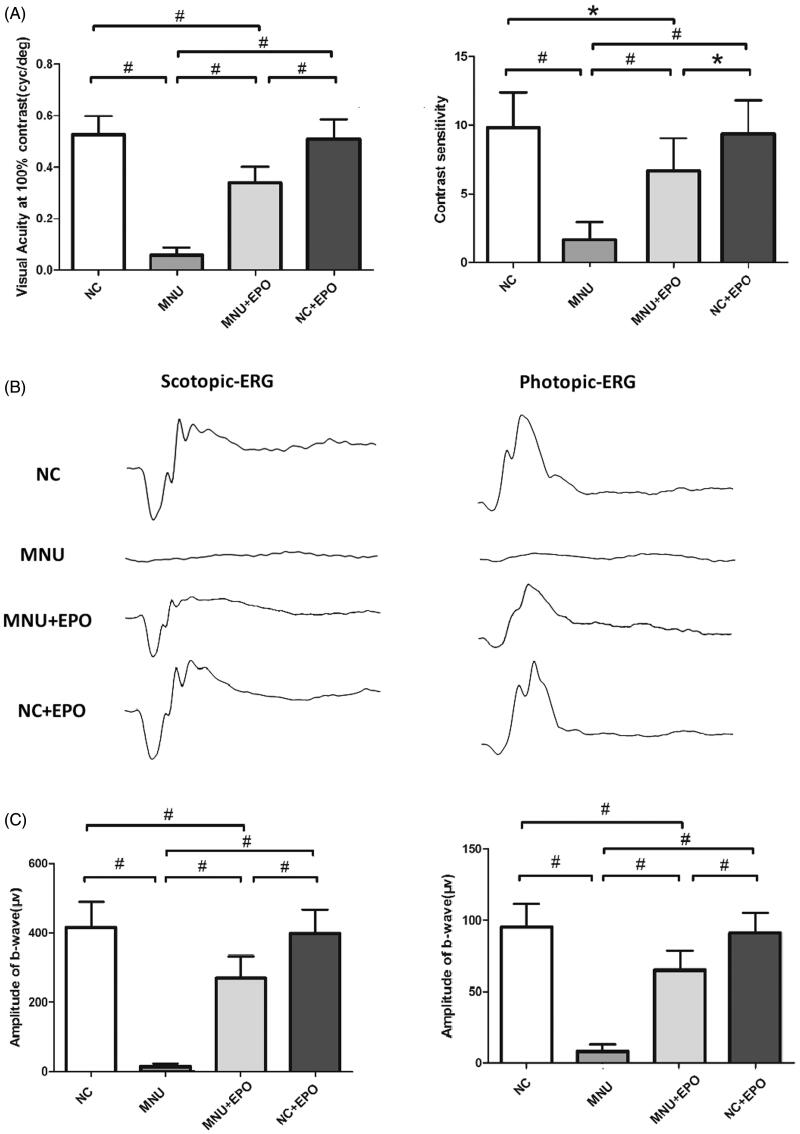
(A) The visual acuity and contrast sensitivity of MNU group were significantly lower than the normal control group. The visual acuity and contrast sensitivity of the EPO treated group were both significantly larger than the MNU group. The visual acuity and contrast sensitivity of the EPO treated group were smaller than the normal control group. Moreover, the visual acuity and contrast sensitivity of the Normal + EPO group were not significantly different from the normal control group. (B) The representative ERG wave forms of each animal group. The MNU administration induced severe impairments to the ERG function of the MNU group. Both the photopic and scotopic b-waves were almost undetectable in the MNU group. (C) The photopic and scotopic b-wave amplitudes of the EPO treated group were larger than the MNU group, respectively. The photopic and scotopic b-wave amplitudes of the Normal + EPO group were smaller than the normal control, but the differences were not statically significant (#*p* < .01, *p < .05, for differences compared between animal groups; All the values were presented as mean ± SD).

The retinal EPO levels of different animal groups were analyzed by ELISA kit. The retinal EPO level of the EPO treated group was significantly higher than the normal controls (*p* < .01; *n* = 10; Figure 1(B)). The retinal EPO level of the EPO treated group was also significantly higher than the MNU group (*p* < .01; *n* = 10). Moreover, the retinal EPO level of the Normal + EPO group was significantly higher than the normal controls (*p* < .01; *n* = 10), suggesting that the subretinal injection could act as an effective pathway to enhance retinal EPO level. Subsequently, the experimental animals were subjected to the optokinetic tests one week after MNU administration. Notably, the mice in the MNU group responded extremely poor to stimulus: both the visual acuity and contrast sensitivity of MNU group were significantly lower than the normal controls (*p* < .01; *n* = 10; Figure 2(A)). On the other hand, the optokinetic performances of the EPO treated group were significantly better than the MNU group. Both the visual acuity and contrast sensitivity of the EPO treated group were both significantly larger than the MNU group (*p* < .01; *n* = 10). However, the visual acuity and contrast sensitivity of the EPO treated group were significantly smaller than the normal controls (visual acuity: *p* < .01; contrast sensitivity: *p* < .05; *n* = 10), suggesting the visual function of the EPO treated mice were not rescued absolutely by EPO treatment. Moreover, the visual acuity and contrast sensitivity of the Normal + EPO group were not significantly different from those of the Normal controls (*p >* .05; *n* = 10) suggesting that the EPO treatment did not affect the optokinetic performances of normal mice. Furthermore, the experimental animals were subjected to ERG examination. The representative ERG waveforms of each animal group are shown in the Figure 2(B). As expected, the MNU toxicity-induced severe impairments on the ERG function of the MNU group. Both the photopic and scotopic b-waves were almost undetectable in the MNU group. Conversely, the EPO treated mice exhibited significantly better ERG function: both the photopic and scotopic b-wave amplitudes of the EPO treated group were larger than the MNU group (*p* < .01; *n* = 10, Figure 2(C)). Moreover, the photopic and scotopic b-wave amplitudes of the Normal + EPO group were smaller than the normal control, but the differences were not statically significant (*p >* .05; *n* = 10). Collectively, these findings suggested the EPO treatment could ameliorate the MNU-induced visual impairments without giving rise to obvious reverse effects.

### Subretinal delivery of EPO alleviated the MNU-induced morphological devastation

**Figure 3.**
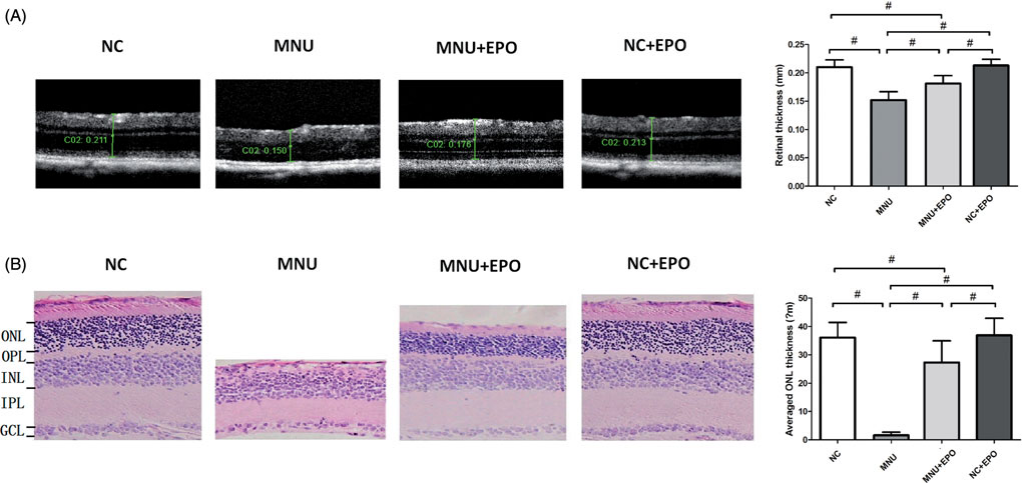
(A) The SD-OCT examination suggested the retinal architecture of MNU group was significantly devastated by MNU administration. The retinal thickness of MNU group was significantly smaller than the normal controls. The retinal thickness of the EPO treated group was smaller than the normal controls. However, the retinal thickness of the EPO treated group was larger than that in the MNU group. The retinal thickness of the normal + EPO group was not significantly different from the normal controls. (B) The ONL of the MNU group disappeared after MNU administration. Conversely, a large proportion of ONL was retained in the retinas of EPO treated group. The mean ONL thickness of the EPO treated group was significantly smaller than the normal controls. However, the mean ONL thickness of the EPO treated group was significantly larger than the MNU group. The mean ONL thickness of the Normal + EPO group was not significantly different from the normal controls (#*p* < .01, for differences compared between animal groups; all the values were presented as mean ± SD; ONL: outer nuclear layer; OPL: outer plexiform layer; INL: inner nuclear layer; IPL: inner plexiform layer; GCL: ganglion cell layer).

The SD-OCT examination was performed to assess the retinal architecture of the experimental animals in vivo (Figure 3(A)). The retinal architecture of the MNU group was severely devastated by MNU administration. The mean retinal thickness of the MNU group was significantly smaller than the normal controls (*p* < .01; *n* = 10). The mean retinal thickness of the EPO treated group was also smaller than the normal controls (*p <* .01; *n* = 10). However, the mean retinal thickness of the EPO treated group was significantly larger than the MNU group (*p <* .01; *n* = 10), indicating that the MNU-induced morphological devastation was partly alleviated by EPO treatment. Particularly, the retinal thickness of the Normal + EPO group was not significantly different from the normal controls (*p >* .05; *n* = 10). No morphological alteration was found in the retinas of Normal + EPO group, suggesting that EPO would not cause pathophysiological changes in mice retinas. In greater detail, the ONL thickness of retinal section was quantified to evaluate the viability of photoreceptors (Figure 3(B)). The ONL in the retinas of the MNU group disappeared after MNU administration. Conversely, a large proportion of ONL was retained in the retinas of EPO treated group. The mean ONL thickness of the EPO group was significantly smaller than the normal controls (*p* < .01; *n* = 10). However, it was significantly larger than the MNU group (*p* < .01; *n* = 10), suggesting the photoreceptors were effectively rescued by EPO. Additionally, the mean ONL thickness of the Normal + EPO group was not significantly different from the normal controls (*p >* .05; *n* = 10).

### EPO-induced protection on the cone photoreceptors of MNU administered mice

**Figure 4.**
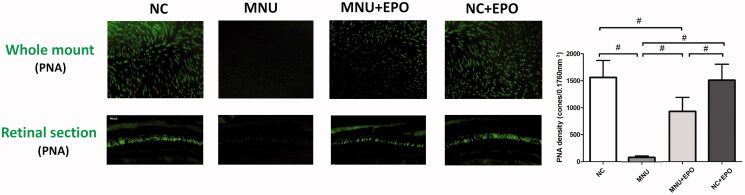
The typical PNA staining was detected in the retinal sections of normal control group. Conversely, no PNA staining was found in the retina sections of the MNU group. The PNA staining could also be detected at the outer segments of EPO treated group. The staining results from the whole mounts agreed well with those from the retinal sections. The PNA staining density of the MNU group was smaller than the normal controls. On the other hand, the PNA staining density of the EPO treated group was significantly larger than the MNU group (#*p* < .01, for differences compared between animal groups; all the values were presented as mean ± SD).

**Figure 5.**
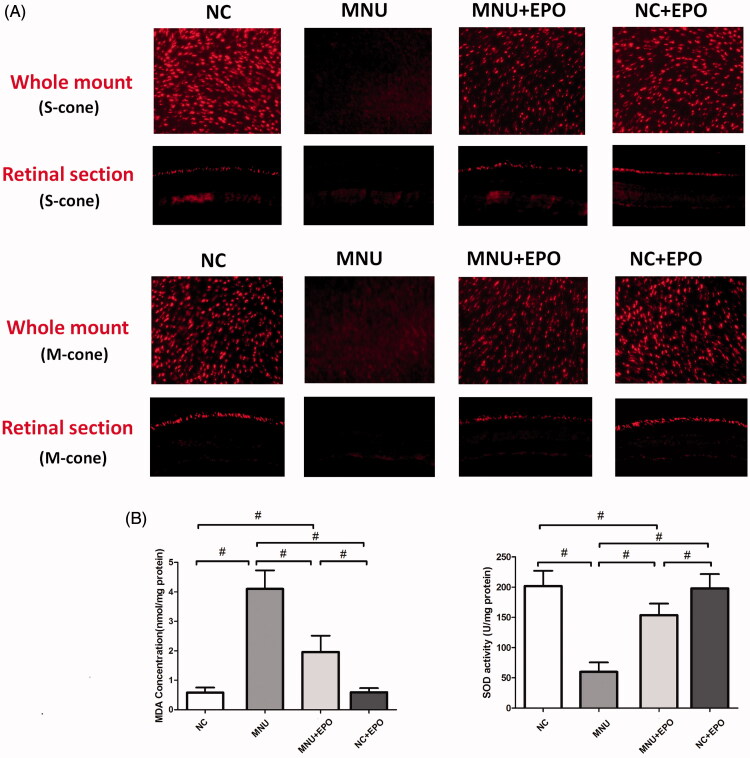
(A) Both the M-opsin and S-opsin staining were detected throughout the retinal section of the EPO treated group, although with a decayed intensity compared with normal control group. Conversely, no M-opsin or S-opsin staining was detected in the retinal section of the MNU group. The immunostaining work based on retinal whole mounts showed a proportion of M-opsin and S-opsin staining was retained in the EPO treated mice. (B) The retinal MDA concentration of the MNU group was significantly higher than the normal controls. The retinal MDA concentration of the EPO treated group was significantly lower than the MNU group. The retinal MDA concentration of Normal + EPO group was not different significantly from normal controls. Meanwhile, the retinal SOD level of EPO treated group was significantly higher than the MNU group (#*p* < .01, for differences compared between animal groups; all the values were presented as mean ± SD).

Typically, rods account for the majority of photoreceptor populations in murine retinas (∼97%). The ONL should be considered as an indicator of rods viability (Szel et al., 1996). Consequently, we performed immunostaining experiments to verify the viability of cones in both retinal sections and whole mounts (Figure 4). In the normal control group, typical PNA staining was detected at the outer segments of retinal sections. Conversely, no PNA staining was found in the retina sections of the MNU group. The PNA staining could also be detected at the outer segments of the EPO treated group. The PNA staining result from the retinal whole mounts were consistent with that from the retinal sections. The PNA staining density of the MNU group was smaller than the normal controls (*p* < .01; *n* = 10). On the other hand, the PNA staining density of the EPO treated group was significantly larger than the MNU group (*p <* .01; *n* = 10). On closer inspection, the M-opsin and S-opsin staining were both detected throughout the retinal section of the EPO treated group, although with a decayed intensity than the normal controls (Figure 5(A)). Conversely, no M-opsin or S-opsin staining was found in the retinal section of the MNU group. The immunostaining study based on retinal whole mounts also suggested a significant proportion of M-opsin and S-opsin staining was retained in the EPO treated mice.

### The mechanisms underlying the EPO-induced protection

**Figure 6.**
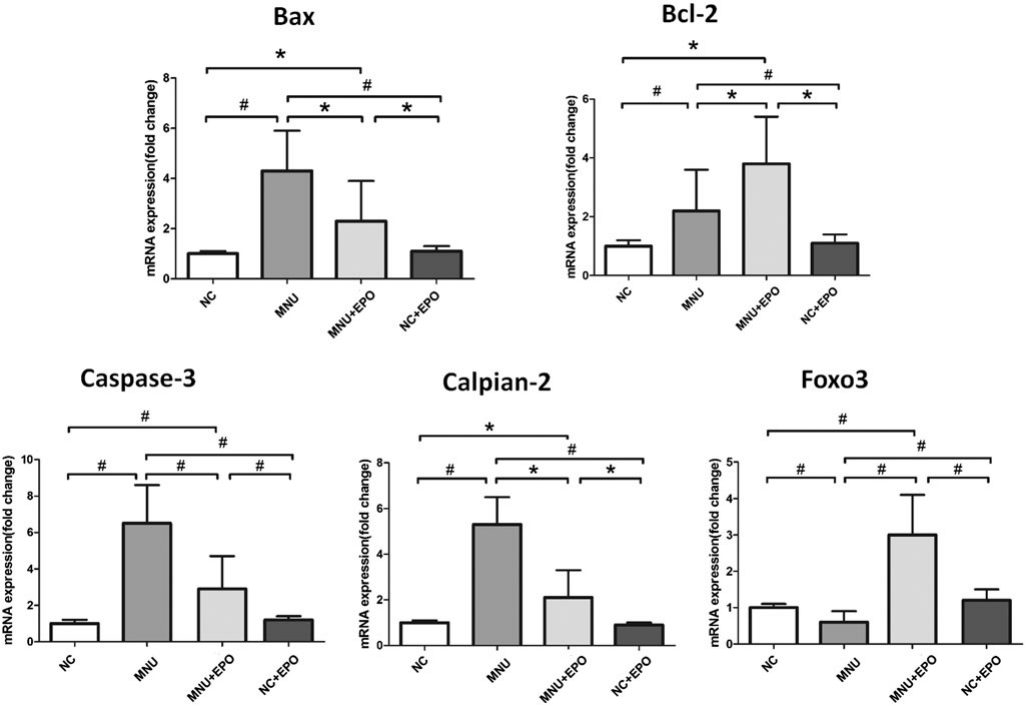
The retinal mRNA levels of Bax, Caspase-3, Bcl-2, and Calpian-2 in the MNU group were significantly higher than the normal controls. On the other hand, the mRNA levels of Bax, Caspase-3, and Calpian-2 in the EPO treated group were significant lower than the MNU group. Additionally, the mRNA level of Bcl-2 and Foxo3 in the EPO treated group was significantly higher than the MNU group, suggesting the anti-apoptotic mechanism contributed to the EPO mediated protection (^#^*p* < .01, *p < .05, for differences compared between animal groups; all the values were presented as mean ± SD).

The retinal MDA concentration of the MNU group was significantly higher than the normal controls (*p* < .01; *n* = 10; Figure 5(B)), suggesting the MNU toxicity-induced excessive lipid oxidation in retinas. However, the retinal MDA concentration of the EPO treated group was significantly lower than the MNU group (*p* < .01; *n* = 10), suggesting that the MNU-induced oxygen stress was alleviated by EPO treatment. Additionally, the retinal MDA concentration of Normal + EPO group was not significantly different from normal controls (*p* > .05; *n* = 10). Subsequently, we examined the SOD level in the retinas of experimental animals. The retinal SOD level of the MNU group was significantly lower than the normal controls (*p* < .01; *n* = 10). Meanwhile, the retinal SOD level of EPO treated group was significantly higher than the MNU group (*p* < .01; *n* = 10), indicating that EPO bolstered the endogenous anti-oxidation system of mouse retinas. Furthermore, the retinal mRNA levels of several classic apoptotic factors were examined by qRT-PCR tests (Figure 6). The retinal mRNA levels of Bax, Caspase-3, Bcl-2, and Calpian-2 in the MNU group were significantly higher than the normal controls (*p* < .01; *n* = 10), suggesting that the apoptotic cascades were activated by MNU administration. On the other hand, the mRNA levels of Bax, Caspase-3, and Calpian-2 in the EPO treated group were significant lower than the MNU group (Bax: *p* < .05; Calpian-2: *p* < .05; Caspase-3: *p* < .01; *n* = 10). Additionally, the mRNA level of Bcl-2 and Foxo3 in the EPO treated group was significantly higher than the MNU group (Bcl-2: *p* < .05; Foxo3: *p* < .01; *n* = 10), suggesting the anti-apoptotic mechanisms correlated with the EPO mediated protection.

## Discussion

In the past decades, more than 180 primary gene mutations have been identified to initiate photoreceptor degeneration in RP. In view of the enormous genetic heterogeneity, a generally applicable treatment would be desirable to halt the progressive photoreceptor loss in a mutation-independent manner (Rivas & Vecino, 2009; Kruk et al., 2015; Geneva II, 2016). EPO is a hematopoietic cytokine that stimulates the proliferation, differentiation, and survival of erythroid progenitor cells. Beyond its well-established hormonal role in modulating erythropoiesis, EPO also functions as an exogenous neuroprotectant against ischemic, toxic, and traumatic insults (Maiese et al., 2004; Juul et al., 2015; Luo et al., 2015). Herein, the subretinal delivery of EPO alleviates photoreceptor degeneration in the MNU administered mouse. Mouse exposed to MNU toxicity has been used as a model to evaluate the beneficial effects of various therapeutic candidates (Tsubura et al., 2010). The MNU administration induces massive photoreceptor degeneration with rapid degenerative dynamics. However, both these morphological devastations and visual functional impairments are ameliorated by EPO treatment. Mechanism studies suggest that these beneficial effects should be ascribed to the anti-apoptosis and anti-oxidative prosperities of EPO. Taken together, our findings highlight the opportunity to develop the EPO into a mutation-independent therapy against RP.

Generally, the systemic administration of EPO would result in the unwanted hematocrit elevation which further exacerbates angiogenesis and thrombosis (Ehrenreich et al., 2009). Moreover, chronic systemic administration of EPO leads to the generation of anti-EPO antibodies and the decreased level of EPO receptors on cellular surface (Maiese et al., 2004). Therefore, we choose the subretinal injection as the delivery pathway of EPO to minimize the unwanted side effects. The subretinal cavity, which located between the RPE cells and the outer segments of photoreceptors, can be efficiently targeted by therapeutic molecular (Pang et al., 2011). The subretinal injection is as a stable, practical, and effective route to delivery reagents in ophthalmologic practice (Timmers et al., 2001; Nour et al., 2003). Herein, we show the subretinal injection of EPO would not give rise to serious detrimental effects on photoreceptor viability. Typically, EPO has a pharmacokinetic profile with faster clearance, which is favorable for episodic intraocular injection (Zhang et al., 2008). A single intravitreal EPO injection in patients with DR is proven to be innocuous. Although future clinical investigations are necessary to analyze potential risks of exogenous EPO in RP patients, our initial evidences suggest the subretinal delivery of EPO could act as a feasible therapeutic strategy. As an invasive delivery method, the subretinal injection would cause transient retinal detachment that yields ERG impairments in subjects. However, the neuroretina layer and RPE layer could reattach within several days post-injection (Nour et al., 2003; Qi et al., 2015). In this study, the b-wave amplitudes of the EPO treated group were still significantly smaller than the normal control group one week after injection. These results suggest that the EPO is not potent enough to eliminate completely the MNU related visual functional impairments and induce a full protection. Moreover, the injection related damages might be the secondary reason for the decreased b-wave amplitudes of the EPO treated group than the normal controls.

Immunostaining experiments allow us to dissect the EPO-induced protective effects on the cone photoreceptors. Generally, the cone photoreceptors are innocent in the RP etiology since all the primary causative mutations occur in the rod-related genes (Shintani et al., 2009). Why the rod-related mutations lose their specificity and lead to the secondary cone death have not been determined yet. A pioneering study shows that the RP animals which are treated with insulin have prolonged cone survival, whereas depletion of endogenous insulin has the opposite effect. These data suggest that the non-autonomous cone death in RP should, at least in part, be a result of the starvation of cones (Punzo et al., 2009). Inasmuch as the cones are primarily responsible for photopic and color vision in human, rescuing cone photoreceptor is recognized as a pivotal component of any successful therapy for RP. As long as the cone photoreceptors are saved by neuroprotectant, RP patients might function well under bright circumstance and carry on relatively normal lives despite the primary rods loss (Mowat et al., 2017). We report for the first time that both the M-cone and S-cone populations in the EPO treated mice are efficiently preserved. Typically, there are two types of cone photoreceptors in mice retinas, with M-cones having a green-sensitive pigment, and S-cones having an ultraviolet (UV)-sensitive pigment. Unlike human retina, in which the cones are exclusively segregated in the central macula (fovea), the cone populations in mice retinas have distinctive regional distribution patterns: S-cones are mainly distributed in the inferior hemisphere, particular the nasal-inferior quadrant; M-cones are mainly distributed in superior hemisphere (Szél et al., 1992; Applebury et al., 2000). Advantageously, the immunostaining experiments based on retinal whole mounts could afford a comprehensive assessment of the of cones viability in different locations. The results harvested from retinal whole mounts are consistent with that form the retinal sections.

Admittedly, the etiology of the MNU-induced photoreceptor degeneration is different from what occurs in RP patients, although the pathologies are somewhat similar. The MNU-induced photoreceptor degeneration is rapidly progressing. However, the RP is a relatively chronic retinopathy in human patients. Therefore, the progression rate should be considered as an instinctive disadvantage of the MNU-induced models, because it deviates somewhat from the natural history of RP. Moreover, the observation that cones are also killed by MNU toxicity does not correspond to cone degeneration in RP patients, since the demise of this population occurs in a secondary wave after rod death. A higher susceptibility of the rod photoreceptors to MNU has been reported in arvicanthis and zebrafish which are both cone-dominant species (Boudard et al., 2010; Tappeiner et al., 2013; Maurer et al., 2014). Therefore, it is highly possible that the cone photoreceptors might be rather protected from the MNU effects than saved by the EPO. Accordingly, a cone-dominant animal model (e.g. Nrl-/- mouse) would be more suitable to study the EPO mediated effects on cone photoreceptors. These discrepancies and limitations of the MNU-induced animal models are crucial to study the EPO mediated effects on cone photoreceptors.

Several lines of evidences suggest the oxidative stress plays a critical role in photoreceptor degeneration (Nowak, 2013; Kruk et al., 2015). As indicated by the elevated MDA concentration, the MNU toxicity results in excessive polyunsaturated lipid peroxidation which would be detrimental to retinal homeostasis. However, the EPO treatment mitigates the MDA concentration while enhances the retinal SOD activity in MNU administered mouse. These findings suggest the EPO ameliorates the retinal oxidative stress in MNU administered mouse (Shen et al., 2014). Typically, Müller cells are the dominating macroglial cells of the vertebrate retina. Müller cells of MNU-treated mice are reactive and respond with a form of gliosis which is characterized by cellular hypertrophy (Reisenhofer et al., 2016). Given the glia cells are the main cellular source of retinal endogenous antioxidants, such as SOD, the activated Müller cell might respond to the MNU impact by increasing their SOD even without the EPO treatment. Therefore, future works should aim at investigating the Müller cell responses and possibly link these activities with the protective effects against retinal degenerations.

Bcl-2 family proteins play important roles in governing the classic apoptotic signal pathway (Levy & Claxton, 2017). They would respond to detrimental stimuli and modulate the release of apoptotic factors. In this context, the retinal neurons might respond to the MNU challenges via an intrinsic adaptive mechanism. It has been shown that the MNU administration increases the expressions of Bcl-2 family proteins (Bcl-xL) in the rat retina (Gao et al., 2010). These results agree well with our findings: the expression level of Bcl-2 increases significantly in the retinas of MNU administered mice. Furthermore, several lines of evidences suggest the over-expression of Bcl-2 causes a long-term inhibition of photoreceptor apoptosis in RP animal models (Eversole-Cire et al., 2000; Nir et al., 2000). In this study, the EPO treatment up-regulates the Bcl-2 expressions in the MNU administered mice, thereby promoting the photoreceptor survival. In the Sprague-Dawley rats, EPO enhanced the expressions of Bcl-xL in retinal neurons through the extracellular signal regulated Kinase (ERK) and Akt pathways (Shen et al., 2010). Whether these mechanisms hold true in the MNU administered mouse remained to be explored by further study. Given the key role of Bcl-2 in maintaining the apoptotic equilibrium, it is highly possible that the expression levels of other apoptotic factors might be also affected by EPO treatment. This notion if further reinforced by the fact that the expression levels of Bax and Caspase-3, two critical pro-apoptotic factors, are both down-regulated after EPO treatment. Collectively, these findings suggest the EPO can rectify the abnormalities in apoptotic cascades. Excessive activation of apoptotic cascades is considered as a forward feeding loop in the photoreceptor degeneration. In this context, a single therapeutic agent with impressive anti-apoptotic attributes has advantageous potentials to combat with heterogeneous RP phenotypes. Particularly, the EPO therapy could reduce the expression level of Calpain-2, a calcium dependent cysteine protease which plays a significant role in photoreceptor degeneration (Paquet-Durand et al., 2007). Calpain activation would suppress the basal autophagy which functions as an essential survival mechanism by producing energy from the breakdown of deleterious products and organelle (Zhou et al., 2015). In contrast, Calpain inhibition could promote photoreceptor survival by restoring basal autophagy (Paquet-Durand et al., 2006; Kuro et al., 2011). Therefore, the EPO-induced may restore Basal autophagy in the mouse retina via inhibiting the Calpain-2 expression. Foxo transcription factor plays a role in cell proliferation and survival by regulating multiple cellular activities including cell cycle arrest, DNA repair, and apoptosis (Myatt & Lam, 2007). Moreover, Foxo is a known regulator of anti-oxidant response and is crucial for maintaining cellular homeostasis. The activation of Foxo transcription factor would alleviate oxidative stress by the induction of antioxidant enzymes, such as catalase and manganese SOD (Ponugoti et al., 2012). A pioneering study suggests the Foxo3 is required for RPE survival at baseline and is also indispensable for the antioxidant-mediated protection (Hanus et al., 2015). Additionally Foxo3 activation can maintain autophagic activities which are essential for photoreceptor survival (Zhou et al., 2015; Shin et al., 2016). Herein, we found the expression level of Foxo is closely associated with the EPO-induced protection, verifying its beneficial role in photoreceptor rescue. Taken together, these findings suggest several mechanisms work concomitantly in the EPO-induced protection.In summary, we show that the subretinal injection could act as an efficient delivery pathway for EPO. This therapeutic strategy could alleviate the MNU-induced photoreceptor degeneration without obvious detrimental effects. The anti-oxidative and anti-apoptotic mechanisms work concomitantly to mediate the EPO-induced protection. These findings would enrich our pharmacological knowledge about EPO and shed light on the development of an effective therapy against RP.

## References

[CIT0001] Applebury ML, Antoch MP, Baxter LC, et al. (2000). The murine cone photoreceptor: a single cone type expresses both S and M opsins with retinalspatial patterning. Neuron 27:513–23. 11055434 10.1016/s0896-6273(00)00062-3

[CIT0002] Boudard DL, Tanimoto N, Huber G, et al. (2010). Cone loss is delayed relative to rod loss during induced retinal degeneration in the diurnal cone-rich rodent Arvicanthis ansorgei. Neuroscience 169:1815–30. 20600653 10.1016/j.neuroscience.2010.06.037

[CIT0003] Caprara C, Grimm C. (2012). From oxygen to erythropoietin: relevance of hypoxia for retinal development, health and disease. Prog Retin Eye Res 31:89–119. 22108059 10.1016/j.preteyeres.2011.11.003

[CIT0004] Ehrenreich H, Weissenborn K, Prange H, EPO Stroke Trial Group, et al. (2009). Recombinant human erythropoietin in the treatment of acute ischemic stroke. Stroke 40:e647–56. 19834012 10.1161/STROKEAHA.109.564872

[CIT0005] Eversole-Cire P, Concepcion FA, Simon MI, et al. (2000). Synergistic effect of Bcl-2 and BAG-1 on the prevention of photoreceptor cell death. Invest Ophthalmol Vis Sci 41:1953–61. 10845622

[CIT0006] Fernández-Sánchez L, Lax P, Noailles A, et al. (2015). Natural compounds from saffron and bear bile prevent vision loss and retinal degeneration. Molecules 20:13875–93. 26263962 10.3390/molecules200813875 PMC6332441

[CIT0007] Gao Y, Deng XG, Sun QN, Zhong ZQ. (2010). Ganoderma spore lipid inhibits N-methyl-N-nitrosourea-induced retinal photoreceptor apoptosis *in vivo*. Exp Eye Res 90:397–404. 20003911 10.1016/j.exer.2009.11.017

[CIT0008] Gawad AE, Schlichting L, Strauss O, Zeitz O. (2009). Antiapoptotic properties of erythropoietin: novel strategies for protection of retinal pigmentepithelial cells. Eye (Lond) 23:2245–50. 19151655 10.1038/eye.2008.398

[CIT0009] Geneva II. (2016). Photobiomodulation for the treatment of retinal diseases: a review. Int J Ophthalmol 9:145–52. 26949625 10.18240/ijo.2016.01.24 PMC4768515

[CIT0010] Hanus J, Zhang H, Chen DH, et al. (2015). Gossypol acetic acid prevents oxidative stress-induced retinal pigment epithelial necrosisby regulating the FoxO3/sestrin2 pathway. Mol Cell Biol 35:1952–63. 25802279 10.1128/MCB.00178-15 PMC4420921

[CIT0011] Juul SE, Mayock DE, Comstock BA, Heagerty PJ. (2015). Neuroprotective potential of erythropoietin in neonates; design of a randomized trial. Matern Health Neonatol Perinatol 1:27. 27057344 10.1186/s40748-015-0028-z PMC4823689

[CIT0012] Kruk J, Kubasik-Kladna K, Aboul-Enein HY. (2015). The role oxidative stress in the pathogenesis of eye diseases: current status and a dual role of physical activity. MRMC 16:241–57. 10.2174/1389557516666151120114605 26586128

[CIT0013] Kuro M, Yoshizawa K, Uehara N, et al. (2011). Calpain inhibition restores basal autophagy and suppresses MNU-induced photoreceptor celldeath in mice. In Vivo 25:617–23. 21709005

[CIT0014] Levy MA, Claxton DF. (2017). Therapeutic inhibition of BCL-2 and related family members. Expert Opin Investig Drugs 26:293–301. 10.1080/13543784.2017.1290078 28161988

[CIT0015] Li W, Sinclair SH, Xu GT. (2010). Effects of intravitreal erythropoietin therapy for patients with chronic and progressive diabeticmacular edema. Ophthalmic Surg Lasers Imaging 41:18–25. 20128565 10.3928/15428877-20091230-03

[CIT0016] Luo W, Hu L, Wang F. (2015). The protective effect of erythropoietin on the retina. Ophthalmic Res 53:74–81. 25592771 10.1159/000369885

[CIT0017] Maiese K, Li F, Chong ZZ. (2004). Erythropoietin in the brain: can the promise to protect be fulfilled? Trends Pharmacol Sci 25:577–83. 15491780 10.1016/j.tips.2004.09.006

[CIT0018] Maurer E, Tschopp M, Tappeiner C, Sallin P, Jazwinska A, Enzmann V. (2014). Methylnitrosourea (MNU)-induced retinal degeneration and regeneration in the zebrafish: histological and functional characteristics. J Vis Exp 20:e51909. 10.3791/51909 PMC4353290 25350292

[CIT0019] Moskowitz A, Hansen RM, Fulton AB. (2016). Retinal, visual, and refractive development in retinopathy of prematurity. Eye Brain 8:103–11. 28539805 10.2147/EB.S95021 PMC5398748

[CIT0020] Mowat FM, Occelli LM, Bartoe JT, et al. (2017). Gene therapy in a large animal model of PDE6A-retinitis pigmentosa. Front Neurosci 11:342. 28676737 10.3389/fnins.2017.00342 PMC5476745

[CIT0021] Myatt SS, Lam EW. (2007). The emerging roles of forkhead box (Fox) proteins in cancer. Nat Rev Cancer 7:847–59. 17943136 10.1038/nrc2223

[CIT0022] Nir I, Kedzierski W, Chen J, Travis GH. (2000). Expression of Bcl-2 protects against photoreceptor degeneration in retinal degeneration slow (rds) mice. J Neurosci 20:2150–4. 10704489 10.1523/JNEUROSCI.20-06-02150.2000 PMC6772515

[CIT0023] Nour M, Quiambao AB, Peterson WM, et al. (2003). P2Y(2) receptor agonist INS37217 enhances functional recovery after detachment caused by subretinal injection in normal and rds mice. Invest Ophthalmol Vis Sci 44:4505–14. 14507899 10.1167/iovs.03-0453 PMC2937827

[CIT0024] Nowak JZ. (2013). Oxidative stress, polyunsaturated fatty acids-derived oxidation products and bisretinoids as potential inducers of CNS diseases: focus on age-related macular degeneration. Pharmacol Rep 65:288–304. 23744414 10.1016/s1734-1140(13)71005-3

[CIT0025] Pang JJ, Dai X, Boye SE, et al. (2011). Long-term retinal function and structure rescue using capsid mutant AAV8 vector in the rd10 mouse, a model of recessive retinitis pigmentosa. Mol Ther 19:234–42. 21139570 10.1038/mt.2010.273 PMC3034861

[CIT0027] Paquet-Durand F, Azadi S, Hauck SM, et al. (2006). Calpain is activated in degenerating photoreceptors in the rd1 mouse. J Neurochem 96:802–14. 16405498 10.1111/j.1471-4159.2005.03628.x

[CIT0026] Paquet-Durand F, Johnson L, Ekstrom P. (2007). Calpain activity in retinal degeneration. J Neurosci Res 85:693–702. 17171699 10.1002/jnr.21151

[CIT0028] Pasantes-Morales H, Quiroz H, Quesada O. (2002). Treatment with taurine, diltiazem, and vitamin E retards the progressive visual field reduction in retinitis pigmentosa: a 3-year follow-up study . Metab Brain Dis 17:183–97. 12322788 10.1023/a:1019926122125

[CIT0029] Petit L, Ma S, Cheng SY, et al. (2017). Rod outer segment development influences AAV-mediated photoreceptor transduction after subretinal injection. Hum Gene Ther 28:464–81. 28510482 10.1089/hum.2017.020 PMC5488363

[CIT0030] Ponugoti B, Dong G, Graves DT. (2012). Role of forkhead transcription factors in diabetes-induced oxidative stress. Exp Diabetes Res 2012:939751. 22454632 10.1155/2012/939751 PMC3290826

[CIT0031] Punzo C, Kornacker K, Cepko CL. (2009). Stimulation of the insulin/mTOR pathway delays cone death in a mouse model of retinitis pigmentosa. Nat Neurosci 12:44–52. 19060896 10.1038/nn.2234 PMC3339764

[CIT0032] Qi Y, Dai X, Zhang H, et al. (2015). Trans-corneal subretinal injection in mice and its effect on the function and morphology of the retina. PLoS One 28:e0136523. 10.1371/journal.pone.0136523 PMC4552822 26317758

[CIT0033] Reisenhofer M, Pannicke T, Reichenbach A, Enzmann V. (2016). Characteristics of Müller glial cells in MNU-induced retinal degeneration. Vis Neurosci 33:E013. 28359347 10.1017/S0952523816000109

[CIT0034] Rivas MA, Vecino E. (2009). Animal models and different therapies for treatment of retinitis pigmentosa. Histol Histopathol 24:1295–322. 19688697 10.14670/HH-24.1295

[CIT0035] Rösch S, Werner C, Müller F, Walter P. (2017). Photoreceptor degeneration by intravitreal injection of N-methyl-N-nitrosourea (MNU) in rabbits: a pilot study. Graefes Arch Clin Exp Ophthalmol 255:317–31. 27866331 10.1007/s00417-016-3531-7

[CIT0036] Shen J, Wu Y, Xu JY, et al. (2010). ERK- and Akt-dependent neuroprotection by erythropoietin (EPO) against glyoxal-AGEs via modulation of Bcl-xL, Bax, and BAD. Invest Ophthalmol Vis Sci 51:35–46. 19628748 10.1167/iovs.09-3544

[CIT0037] Shen W, Chung SH, Irhimeh MR, et al. (2014). Systemic administration of erythropoietin inhibits retinopathy in RCS rats. PLoS One 13:e104759. 10.1371/journal.pone.0104759 PMC4132022 25119659

[CIT0038] Shin HR, Kim H, Kim KI, Baek SH. (2016). Epigenetic and transcriptional regulation of autophagy. Autophagy 12:2248–9. 27487449 10.1080/15548627.2016.1214780 PMC5103355

[CIT0039] Shintani K, Shechtman DL, Gurwood AS. (2009). Review and update: current treatment trends for patients with retinitis pigmentosa. Optometry 80:384–401. 19545852 10.1016/j.optm.2008.01.026

[CIT0040] Szel A, Rulich P, Caffe AR, Veen van T. (1996). Distribution of cone photoreceptors in the mammalian retina. Microsc Res Tech 35:445–62. 9016448 10.1002/(SICI)1097-0029(19961215)35:6<445::AID-JEMT4>3.0.CO;2-H

[CIT0041] Szél A, Röhlich P, Caffé AR, et al. (1992). Unique topographic separation of two spectral classes of cones in the mouse retina. J Comp Neurol 325:327–42. 1447405 10.1002/cne.903250302

[CIT0042] Tao Y, Chen T, Liu B, et al. (2016). The transcorneal electrical stimulation as a novel therapeutic strategy against retinal and optic neuropathy: a review of experimental and clinical trials. Int J Ophthalmol 9:914–9. 27366697 10.18240/ijo.2016.06.21 PMC4916152

[CIT0043] Tao Y, Chen T, Liu B, et al. (2015). The neurotoxic effects of N-methyl-N-nitrosourea on the electrophysiological property and visual signal transmission of rat's retina. Toxicol Appl Pharmacol 1:44–52. 10.1016/j.taap.2015.03.013 25796171

[CIT0044] Tappeiner C, Balmer J, Iglicki M, et al. (2013). Characteristics of rod regeneration in a novel zebrafish retinal degeneration model using N-methyl-N-nitrosourea (MNU). PLoS One 8:e71064. 23951079 10.1371/journal.pone.0071064 PMC3741320

[CIT0045] Timmers AM, Zhang H, Squitieri A, Gonzalez-Pola C. (2001). Subretinal injections in rodent eyes: effects on electrophysiology and histology of rat retina. Mol Vis 7:131–7. 11435999

[CIT0046] Tsubura A, Yoshizawa K, Kuwata M, Uehara N. (2010). Animal models for retinitis pigmentosa induced by MNU; disease progression,mechanisms and therapeutic trials. Histol Histopathol 25:933–44. 20503181 10.14670/HH-25.933

[CIT0047] Umino Y, Solessio E, Barlow RB. (2008). Speed, spatial, and temporal tuning of rod and cone vision in mouse. J Neurosci 28:189–98. 18171936 10.1523/JNEUROSCI.3551-07.2008 PMC2847259

[CIT0048] Yu DY, Cringle SJ. (2005). Retinal degeneration and local oxygen metabolism. Exp Eye Res 80:745–51. 15939030 10.1016/j.exer.2005.01.018

[CIT0049] Zhang JF, Wu YL, Xu JY, et al. (2008). Pharmacokinetic and toxicity study of intravitreal erythropoietin in rabbits. Acta Pharmacol Sin 29:1383–90. 18954534 10.1111/j.1745-7254.2008.00885.x

[CIT0050] Zhong Y, Li J, Wang JJ, et al. (2012). X-Box binding protein 1 is essential for the anti-oxidant defense and cell survival in the retinal pigment Epithelium. PLoS One 7:e38616. 22715395 10.1371/journal.pone.0038616 PMC3371004

[CIT0051] Zhou Z, Doggett TA, Sene A, et al. (2015). Autophagy supports survival and phototransduction protein levels in rod photoreceptors. Cell Death Differ 22:488–98. 25571975 10.1038/cdd.2014.229 PMC4326583

